# CILP2 overexpression correlates with tumor progression and poor prognosis in patients with colorectal cancer in The Cancer Genome Atlas (TCGA) study

**DOI:** 10.1186/s12957-020-02049-6

**Published:** 2020-10-24

**Authors:** Feng Huang, Yuanfei Peng, Qing Ye, Jinhu Chen, Yangming Li, Shengyuan Liu, Yangmei Xu, Lijie Huang

**Affiliations:** 1 Department of Gastrointestinal Tumor Surgery, Fujian Cancer Hospital & Fujian Medical University Cancer Hospital, Fujian Province Key Laboratory of Tumor Biotherapy, No. 420 Fuma Road, Fuzhou, ,350014 Fujian Province People’s Republic of China; 2 Department of Liver Surgery, Liver Cancer Institute, Zhongshan Hospital, Fudan University, Key Laboratory of Carcinogenesis and Cancer Invasion of Ministry of Education, Shanghai, 200032 People’s Republic of China

**Keywords:** CILP2, Colorectal cancer, TCGA, Immunohistochemistry, Prognosis

## Abstract

**Background:**

Genetic alterations play an important role in the progression of colorectal cancer (CRC). Identifying new biomarkers to assess the prognosis of patients with CRC is critical. Cartilage intermediate layer protein 2 (CILP2) gene, screened from TCGA database by bioinformatics, may be closely related to the progression of CRC. CILP2 was barely reported with clinical features of tumors.

**Materials and methods:**

Clinical information and RNA-seq data were derived from TCGA colorectal carcinoma cohort. CILP2 expression at mRNA level was estimated by bioinformatical analysis of TCGA cases. Tissue microarray (TMA) was constructed containing paraffin-embedded 64 pairs of CRC and matched adjacent normal tissues. The expression at the protein level was detected in 64 pairs of CRC and matched adjacent normal tissues by immunohistochemical analysis. CILP2 expression level and its clinical value were estimated by bioinformatical analysis with linear and logistic regression. Survival analysis was performed between high and low groups of CILP2 expression by Cox regression analysis, and the *P* value was calculated by the log-rank test. The Kaplan-Meier curves were tested by the log-rank test.

**Results:**

CILP2 was statistically significantly higher expressed in the CRC tissues when compared with paired adjacent normal tissues in TCGA cohort (*P* < 0.001) and in the TMA cohort (*P* = 0.001). Also, CILP2 high expression was strongly correlated with T3/4 stage (*P* = 0.001), N1/2/3 stage (*P* = 0.005), M1 stage (*P* = 0.048), and higher clinical stage (UICC 2010 stage) (*P* < 0.001) in TCGA cohort, and also positively associated with T3/4 stage (*P* = 0.022) and higher clinical stage (UICC 2010 stage) (*P* = 0.03) in TMA cohort. Furthermore, CILP2 overexpression predicted poor prognosis and could be an independent prognostic factor (*P* = 0.003).

**Conclusion:**

We revealed that CILP2 is associated with advanced stages and could play a role as an independent predictor of poor survival in CRC.

## Introduction

Colorectal cancer (CRC) is one of the most common cancers that ranks second in cancer-associated mortality in the world, with increasing morbidity in recent years. It was estimated that in 2018, more than 1.8 million new cases occurred with 881,000 deaths [1]. CRC is caused by a variety of factors and is involved in the successive accumulation of genetic and epigenetic alternations [2]. Surgical resection is the mainstay for the treatment of CRC, but tumor recurrence is common. A large cohort study indicated that the median survival time was 13.3 months before recurrence [3]. Numerous works have been done to reveal the underlying mechanisms of CRC, and encouraging progress has been made [4–7]. However, further investigating works are still needed to deeply understand the molecular mechanisms, and molecular biomarkers for both early detection and prognosis are to be developed for better therapeutic uses in the patients.

The Cancer Genome Atlas (TCGA) is a comprehensive cancer research project initiated by the National Cancer Institute (NCI) and the National Human Genome Research (NHGRI). By applying genome analysis technology, especially the large-scale genome sequencing technology, TCGA draws the map of 33 kinds of multiple human tumor genome variation, including 631 CRC samples. We screened the CLIP2 gene from TCGA CRC database by bioinformatics techniques. CILP2, highly cognate with CILP1, is a secreted glycoprotein that has been first isolated from human articular cartilage. Mesenchymal protein (CILPs) plays an important role in cartilage support and is associated with the occurrence of osteoarthritis. A significant association was also highlighted between polymorphisms in the CILP gene and osteoarthritis progression [8]. The *CILP* gene is located on chromosome 19p13. It was reported that through genome-wide association studies (GWAS), the *neurocan-cartilage intermediate layer protein 2-pre-B cell leukemia homeobox 4* (*NCAN-CILP2-PBX4*) region, an intergenic region spanning 300 kb, is associated with concentrations of low-density lipoprotein cholesterol and triglycerides in sera [9]. Eleven genes and one miRNA are encoded in this region. This region has been shown to be consistent and has deep association with serum lipid levels in subsequent studies for individuals of European and Chinese descents [10–12]. In addition to plasma lipid levels, the genome region around *CILP2* was identified as a non-alcoholic fatty liver disease (NAFLD)-associated locus by GWAS in individuals of European descent [13], but not in Japanese individuals [14].

Tumors have been considered as high demands of energy and abnormal anabolism for their rapid growth. In recent years, many studies have found that tumor development is often accompanied by metabolic abnormalities, and lipid mass spectrometry and lipid metabolism abnormalities play a particularly important role in this process [15]. But subtle mechanisms underlying over-reacted lipid metabolism remain poorly understood. In the preliminary study of TCGA and our cohort analysis, we found that CLIP2 gene showed a tendency of up-expression in CRC tissues compared with other genes screened by bioinformatics from TCGA CRC database. Besides, few reports have described the relationship between CILP2 and cancers, except one that reported an expression quantitative trait locus, namely rs8103992, was statistically significantly associated with osteosarcoma risk [16]. In this study, we evaluated CILP2 expression and its correlations with clinicopathological characteristics, such as tumor stages, and overall survival of CRC patients in TCGA, and furtherly verified using immunohistochemistry assay within human CRC tissues, which may provide a new potential molecular marker for prognostic use of the patients.

## Materials and methods

### TCGA data mining and gene expression datasets

The CRC cohort in TCGA was downloaded, and level 3 RNA-seq V2 datasets were used, which was based on Illumina HiSeq 2000. Matched clinical data from CRC patients were also downloaded (https://portal.gdc.cancer.gov/). In the cohort, 621 patients were included, and 609 among them had intact survival data recorded. So, 609 patients were included in the survival analysis in the study. For each gene, the transcript with the highest expression was selected for the following process. Meanwhile, the data of one gene was considered invalid when raw counts of the gene in all samples were less than 50. All filtered gene expressions had been processed and been normalized by trimmed mean of *M* values analysis.

### Tissue microarray construction

Tissue microarray (TMA) construction was carried out as described previously [17]. Briefly, 64 pairs of CRC and matched adjacent normal tissues were obtained from patients undergoing surgery between January 2016 and October 2019 at the Department of Gastrointestinal Tumor Surgery, Fujian Cancer Hospital. TMA recipient block was constructed containing paraffin-embedded 64 pairs of tumor and matched adjacent normal tissues previously fixed in 10% formaldehyde. The most representative tumor or normal areas were carefully selected and marked based on the matched hematoxylin-eosin-stained slides. Altogether, 128 cores (diameter 1.8 mm) of test tissue were taken from the donor blocks with the tissue microarrayer (Beecher Instruments, Silver Spring, MD, USA) and inserted into the recipient block.

### Immunohistochemistry analysis

Immunohistochemistry was carried out as described previously [17]. Briefly, unstained 4-mm sections were cut from the TMA recipient block and deparaffinized in xylene, and the slides were bathed in 0.01 mol/l sodium citrate and heated in a microwave oven for 12 min. The sections were incubated with anti-CILP2 antibody (Santa Cruz, CA, USA) and kept at 4 °C overnight. Negative control slides were treated with only non-immunized mouse immunoglobulin fraction under equivalent conditions. For the secondary developing reagents, a labeled streptavidin-biotin kit (Dako, CA, USA) was used. Slides were developed with diaminobenzaminidine and counterstained with hematoxylin.

### Evaluation of immunostaining results

Immunohistochemistry staining was scored as described previously blindly by two independent pathologists without knowledge of the patient’s clinicopathology and clinical outcome [18, 19]. Positive cases were defined by the presence of intracellular staining with red/brown color in epithelial cells. The expression level of CILP2 was evaluated semi-quantitatively according to the proportion of positively stained tumor cells for CILP2 and the intensity of the staining. The staining intensity ranged between 0 and 3 as follows: (I) 0, no recognizable staining, referred to as negative (−); (II) 1, slight staining, referred to as weakly positive (+); (III) 2, moderate staining, referred to as moderately positive (++); and (IV) 3, distinct staining, referred to as strongly positive (+++).The staining percentage was evaluated as follows: 0**–**10% = 1, 11**–**50 % = 2, 50**–**75% = 3, and 75**–**100% = 4. The immune scores of each patient were calculated by the addition of intensity and percentage together. According to the protein of CILP2 expression, samples were divided into two groups, immune scores greater than or equal to 4 were defined as high expression, and immune scores less than 4 were defined as low expression.

### Statistical analysis

Statistical analyses were performed using SPSS 22.0 software. CILP2 gene expression in different groups (divided by each parameter) was compared using the Mann-Whitney *U* test. Correlation between CILP2 gene expression and different TNM stages was analyzed by Spearman’s test, and Spearman’s rank correlation coefficient (*r*_*s*_) was used to evaluate the strength of association. CILP2 gene expression in different groups was analyzed by one-way ANOVA followed by Welch’s *t* test. CILP2 protein expression in different groups was analyzed by Fisher’s exact test. Survival analysis was performed between high and low groups of CILP2 expression (defined by the median value of CILP2 expression) by Cox regression analysis, and the *P* value was calculated by the log-rank test. The Kaplan-Meier curves were tested by the log-rank test.

## Results

### CILP2 was overexpressed in CRC

**Fig. 1:**
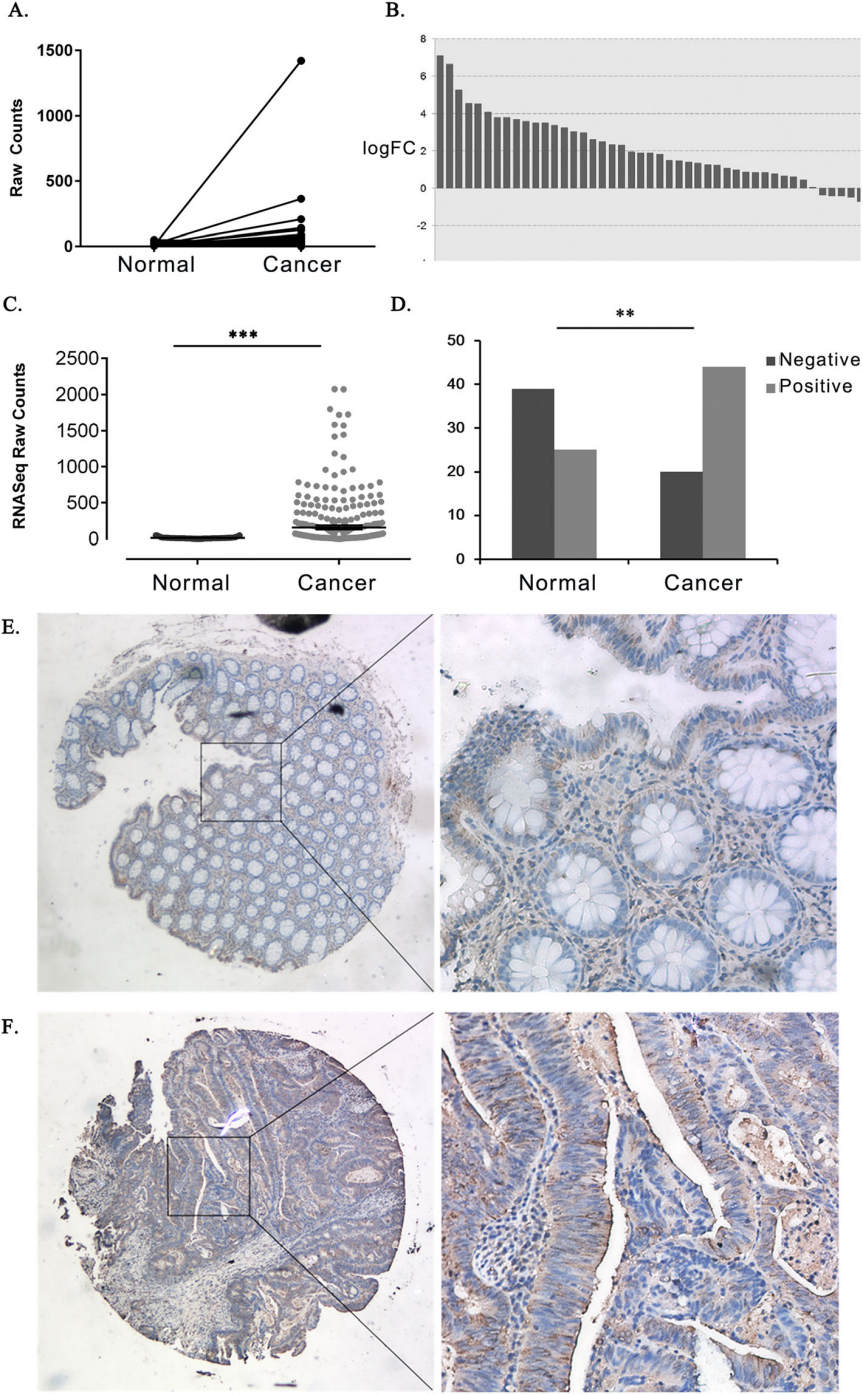
CILP2 was upregulated in colorectal cancer samples. **a**, **b** The line chart and histogram of CILP2 gene expression in 50 paired tumor and matched adjacent normal tissue samples in TCGA cohort. FC, fold change. **c** Scatter plot of CILP2 expression in all normal and cancer samples in TCGA cohort. ***P < 0.001. **d** Positive or negative expression of CILP2 protein in matched adjacent normal tissues (normal) or cancer tissues in the TMA cohort. **P = 0.001. **e** Representative image of normal tissue immunohistochemical staining in the TMA cohort. Left: original magnification × 100; right: original magnification × 400. **f** Representative image of cancer tissue immunohistochemical staining in the TMA cohort. Left: original magnification × 100; right: original magnification × 400

Aiming at searching for potential novel prognostic markers of CRC, we firstly analyze expression data of TCGA CRC cohort from Illumina HiSeq 2000 platform, which contains 621 samples and correlating clinical and demographic information. We found that CILP2 was strongly correlated with clinical features of the samples in TCGA cohort, and CILP2 has not been reported in CRC before. So we focused on CILP2 and furtherly analyzed the association between CILP2 expression and CRC prognosis. To determine the role of CILP2 in CRC, we first analyzed CILP2 gene expression in 50 patients’ samples with paired adjacent normal tissues in TCGA cohort, and the results suggested that CILP2 gene was overexpressed statistically significant in tumor samples compared to paired adjacent normal tissues (Fig. 1a, b; fold change = 3.412; *P* < 0.001; Table 1). Additionally, CILP2 gene expression was upregulated in the total amount of tumor samples compared with adjacent normal tissue samples in TCGA cohort (Fig. 1c, fold change = 8.6161, *P* < 0.001, Table 1).

**Table 1:** CILP2 gene expression in 50 paired tumor and matched adjacent normal tissue samples, and in all normal and cancer samples in TCGA cohort

	ID	Gene symbol	FC	***P*** value	Total sample	Sample unchanged	Sample up	Sample down
**Paired samples**	148113	CILP2	3.412	2.56E−07	50	15	31	4
**Total samples**	148113	CILP2	8.6161	1.28E−19	621	–	–	–

To further testify the upregulation of CILP2 in CRC, we detected CILP2 protein expression in a TMA (*n* = 64) by immunohistochemical staining (IHC). We found that 68.75% (44/64) of tumor tissues highly expressed CILP2 protein, whereas only 39.06% (25/64) of matched adjacent normal tissues highly expressed CILP2 protein. The staining result in our cohort showed that CILP2 protein expression was statistically significantly more prevalent in tumors than in matched adjacent normal tissues (Fig. 1d, *P* = 0.001). The representative images of CILP2 immunostaining are shown in Fig. 1e, f.

### Correlations between CILP2 expression and clinicopathological parameters in CRC

**Fig. 2:**
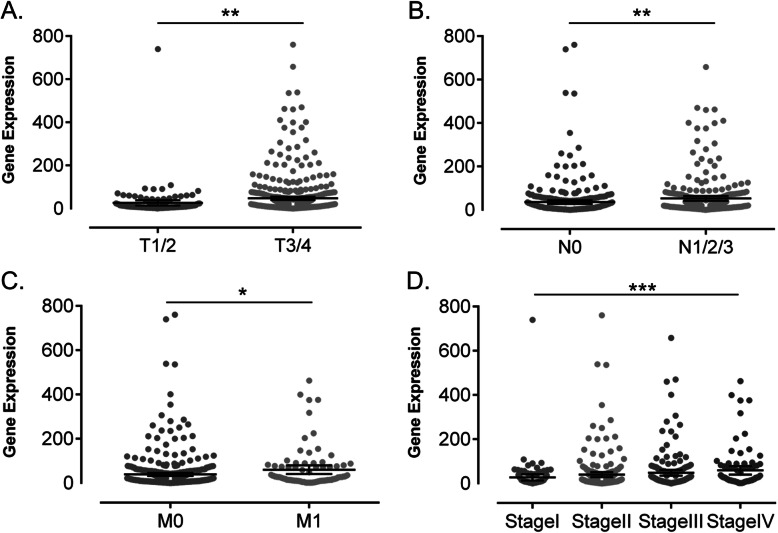
CILP2 expression was correlated with different clinicopathological parameters of CRC patients in TCGA cohort, as in **a** T stages, **b** regional lymph node metastatic patients, **c** distant metastatic patients, and **d** UICC clinical stages. *P < 0.05; **P < 0.01; ***P < 0.001

Furthermore, to dissect the role of CILP2 in CRC carcinogenesis, correlations between CILP2 expression and clinicopathological parameters were analyzed based on TCGA cohort (mRNA) and TMA cohort (protein), presented in Table 2. And Table 3 shows the correlation analysis results of TCGA cohort using Spearman’s test. Among 621 samples from TCGA cohort, part of the clinicopathological data was missed in some cases. Median expression of CILP2 of all samples was chosen as a cutoff to divide samples into the CILP2-high (*n* = 310) group and CILP2-low (*n* = 311) group. We observed that in TCGA cohort, tumors of high CILP2 expression were positively associated with the T3/4 stage (T1/2, 36.51%; T3/4, 53.35%; *P* = 0.001, Table 2) and with the N1/2/3 stage (N0, 44.89%; N1/2/3, 74.29%; *P* = 0.005, Table 2). Similarly, the percentage of tumors with high CILP2 expression increased with grading of clinical stage (UICC 2010 stage) (stage I, 34.29%; stage II, 49.35%; stage III, 54.19%; stage IV, 60.00%; *P* < 0.001, Table 2) and distant metastasis (M0, 48.69%; M1, 60.23%; *P* = 0.048, Table 2). It was suggested that CILP2 gene expression was strongly correlated with T stage (*P* = 0.001), N stage (*P* = 0.005), M stage (*P* = 0.048), and higher clinical stage (*P* < 0.001), respectively, in TCGA cohort (Fig. 2). However, there was no significant correlation of CILP2 expression with patients’ age or gender (*P* > 0.05, Table 2). In TMA cohort, tumors of positive CILP2 protein expression was statistically significantly associated with T3/4 stage (T1/2, 44.83%; T3/4, 74.29%; *P* = 0.022, Table 2) and higher clinical stage (UICC 2010 stage) (stage I, 35.71%; stage II, 50.00%; stage III, 70.83%; stage IV, 90.00%; *P* = 0.03, Table 2). However, there was no significant correlation of CILP2 protein expression with N stage (*P* = 0.2, Table 2); it may be due to a limited set of data. Although the significant correlation was also not reached in our cohort, there was a tendency that tumors with positive CILP2 protein expression were more likely to distantly metastasize compared with CILP2-negative tumors (*P =* 0.074, Table 2).

**Table 2:** Association between CILP2 expression level and clinicopathological parameters in CRC patients

Clinicopathological parameters	Expression of CILP2 mRNA in TGCA			Expression of CILP2 protein in TMA cohort		
	High (*n* = 310)	Low (*n* = 311)	*P* value	High (*n* = 39)	Low (*n* = 25)	*P* value
Age (years)			0.059			0.37
≤ 68 (*n* = 331)	177 (53.47%)	154 (46.53%)		20 (66.67%)	10 (33.33%)	
> 68 (*n* = 290)	133 (45.86%)	157 (54.14%)		19 (55.88%)	15 (44.12%)	
Gender			0.400			0.8
Male (*n* = 331)	160 (48.34%)	171 (51.66%)		22 (62.86%)	13 (37.14%)	
Female (*n* = 290)	150 (51.72%)	140 (48.28%)		17 (58.62%)	12 (41.38%)	
Pathological T stage^a^			**0.001**			**0.022**
T1/2 (*n* = 126)	46 (36.51%)	80 (63.49%)		13 (44.83%)	16 (55.17%)	
T3/4 (*n* = 493)	263 (53.35%)	230 (46.65%)		26 (74.29%)	9 (25.71%)	
N stage^a^			**0.005**			0.2
N0 (*n* = 352)	158 (44.89%)	194 (55.11%)		16 (51.61%)	15 (48.39%)	
N1/2/3 (*n* = 265)	149 (56.23%)	116 (43.77%)		23 (69.70%)	10 (30.30%)	
M stage^a^			**0.048**			0.074
M0 (*n* = 458)	223 (48.69%)	235 (51.31%)		30 (55.56%)	24 (44.44%)	
M1 (*n* = 88)	53 (60.23%)	35 (39.77%)		9 (90.00%)	1 (10.00%)	
Clinical stage^a^			**< 0.001**			**0.03**
Stage I (*n* = 105)	36 (34.29%)	69 (65.71%)		5 (35.71%)	9 (64.29%)	
Stage II (*n* = 229)	113 (49.35%)	116 (50.65%)		8 (50.00%)	8 (50.00%)	
Stage III (*n* = 179)	97 (54.19%)	82 (45.81%)		17 (70.83%)	7 (29.17%)	
Stage IV (*n* = 90)	54 (60.00%)	36 (40.00%)		9 (90.00%)	1 (10.00%)	

*P* value < 0.05 was considered statistically significant (in bold). *T* tumor, *N* regional lymph node, *M* metastasis

^a^Some missing data for parameter

**Table 3:** Correlation between CILP2 expression and TNM stages in CRC patients in TCGA cohort

		CILP2 expression	T stage	N stage	M stage	Clinical stage
CILP2 expression	*r*_*s*_^2^	1.000	0.136	0.112	0.085	0.149
	*P* (two-tailed)	N.A	**0.001**	**0.005**	**0.048**	**< 0.001**
T stage	*r*_*s*_^2^	0.136	1.000	0.308	0.197	0.589
	*P* (two-tailed)	**0.001**	N.A	**< 0.001**	**< 0.001**	**< 0.001**
N stage	*r*_*s*_^2^	0.112	0.308	1.000	0.418	0.846
	*P* (two-tailed)	**0.005**	**< 0.001**	N.A	**< 0.001**	**< 0.001**
M stage	*r*_*s*_^2^	0.085	0.197	0.418	1.000	0.665
	*P* (two-tailed)	**0.048**	**< 0.001**	**< 0.001**	N.A	**< 0.001**
Clinical stage	*r*_*s*_^2^	0.149	0.589	0.846	0.665	1.000
	*P* (two-tailed)	**< 0.001**	**< 0.001**	**< 0.001**	**< 0.001**	N.A

*P* value < 0.05 was considered statistically significant (in bold). *T* tumor, *N* regional lymph node, *M* metastasis

### High CILP2 expression is associated with poor outcome of CRC patients

**Fig. 3:**
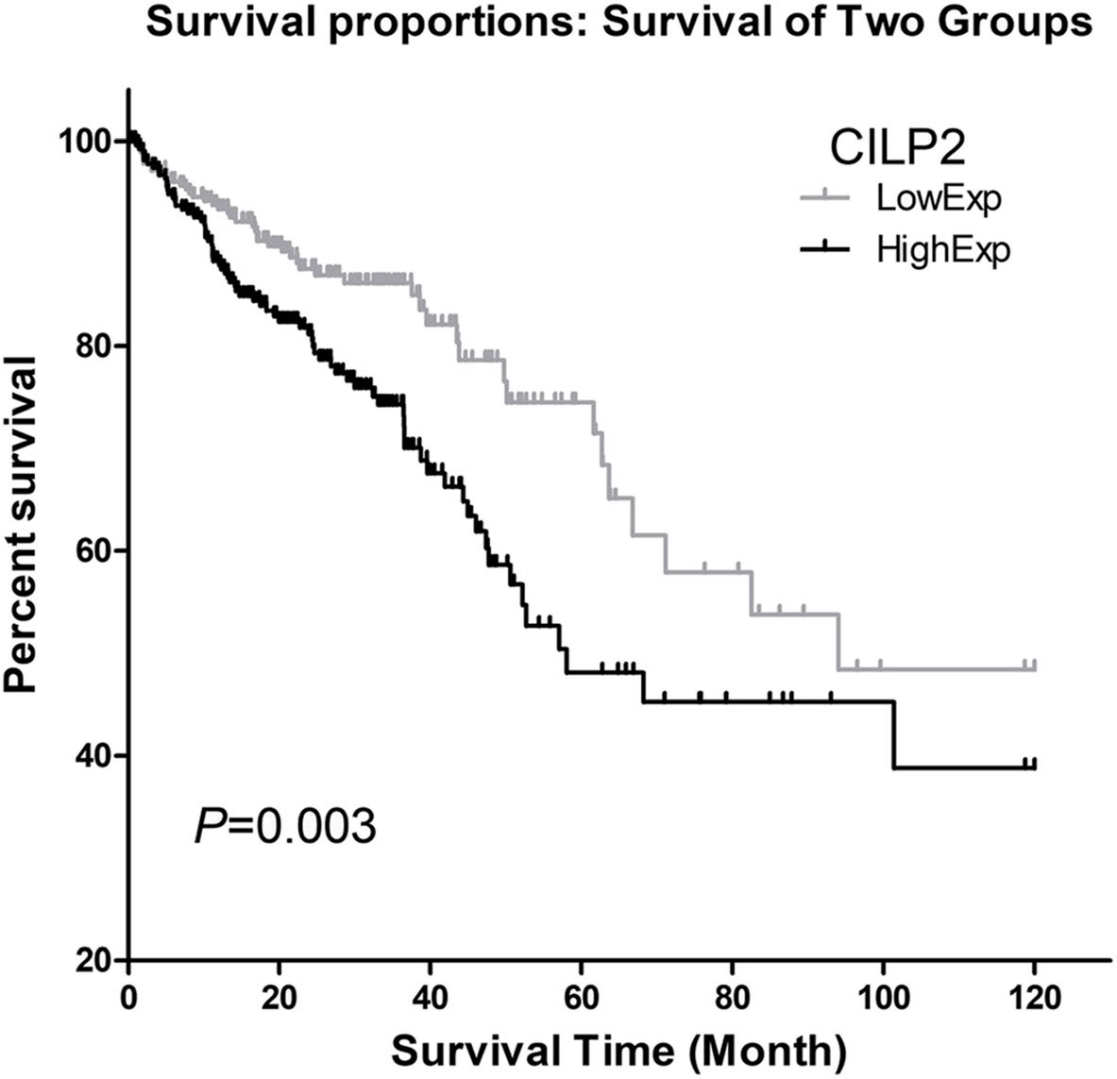
The Kaplan-Meier analysis of CILP2 expression and overall survival in total CRC samples of 10 years in TCGA cohort. Higher CILP2 expression group had a poorer overall survival than the low CILP2 expression group (*P* = 0.003)

The Kaplan-Meier analysis was performed to investigate the relationship between CILP2 expression and overall survival in TCGA cohort. There were 609 CRC samples available for prognostic information. Median expression of CILP2 of all samples was chosen as a cutoff to divide samples into the CILP2-high (*n* = 305) group and CILP2-low (*n* = 304) group. As shown in Fig. 3 and Table 4, CRC patients with high CILP2 expression exhibited a poorer overall survival rate compared with the low-expression group (*P* = 0.003). Moreover, the univariate Cox regression analysis indicated that high CILP2 expression was strongly associated with a poor prognosis (*P* = 0.003). Other clinical variables, such as age (*P* < 0.0001), T stage (*P* = 0.005), N stage (*P* < 0.0001), M stage (*P* < 0.0001), and clinical stage (UICC 2010 stage) (*P* < 0.0001), were all associated with overall survival (Table 5). Moreover, the multivariate analysis revealed that high CILP2 expression (*P* = 0.034), age (*P* < 0.0001), M stage (*P* < 0.0001), and clinical stage (UICC 2010 stage) (*P* = 0.017) were independently associated with a poor prognosis (Table 5).

**Table 4:** Survival analysis was performed by the Kaplan-Meier method

Variables		*N*	Means for survival time (month)	Survival time (month, 95% CI)		*P* value
CILP2 expression	Low	304	83.770	74.181	93.359	**0.003**
	High	305	70.805	61.802	79.807	
	Total	609	77.193	70.583	83.803	
Gender	Male	329	74.017	64.871	83.163	0.941
	Female	280	80.242	70.918	89.566	
	Total	609	77.193	70.583	83.803	
Age	≤ 68	324	86.777	77.219	96.334	**< 0.0001**
	> 68	285	68.173	59.326	77.021	
	Total	609	77.193	70.583	83.803	
T stage	T1/2	126	97.409	81.150	113.667	**0.003**
	T3/4	481	74.489	67.519	81.460	
	Total	607	77.572	70.950	84.194	
N stage	N0	348	88.348	79.810	96.887	**< 0.0001**
	N1/2/3	257	63.017	53.234	72.799	
	Total	605	77.530	70.908	84.152	
M stage	M0	450	87.130	79.689	94.571	**< 0.0001**
	M1	86	37.131	29.468	44.794	
	Total	536	78.778	71.809	85.747	
Clinical stage	Stage I/II	330	89.089	80.335	97.843	**< 0.0001**
	Stage III/IV	262	64.632	54.851	74.413	
	Total	592	78.325	71.646	85.005	

*P* value < 0.05 was considered statistically significant (in bold). *T* tumor, *N* regional lymph node, *M* metastasis

**Table 5:** Survival analysis was performed by univariate and multivariate Cox regression analysis

Variables	Univariate analysis			Multivariate analysis		
	*P* value	HR	95% CI	*P* value	HR	95% CI
CILP2 expression (high vs. low)	0.003	1.713	1.194–2.457	**0.034**	1.547	1.033–2.317
Gender (male vs. female)	0.941	0.987	0.694–1.404	0.132	1.351	0.913–1.999
Age (> 68 vs. ≤ 68)	**< 0.0001**	1.923	1.337–2.767	**< 0.0001**	2.626	1.743–3.957
T stage (T3/4 vs. T1/2)	**0.005**	2.457	1.320–4.572	0.143	1.817	0.816–4.044
N stage (N1/2/3 vs. N0)	**< 0.0001**	2.734	1.897–3.942	0.264	0.589	0.232–1.491
M stage (M1 vs. M0)	**< 0.0001**	4.126	2.776–6.133	**< 0.0001**	2.854	1.769–4.604
Clinical stage (III/IV vs. I/II)	**< 0.0001**	3.	2.046–4.401	**0.017**	3.408	1.244–9.337

*P* value < 0.05 was considered statistically significant (in bold). *T* tumor, *N* regional lymph node, *M* metastasis, *HR* hazard ratio, *CI* confidence interval

## Discussion

The incidence of CRC has risen sharply in recent years [1], with limited diagnostic and prognostic tools for early detection and patients’ survival prediction. Many researches are focusing on the issue, and numerous advances have been achieved to reveal the underlying mechanisms of cancer development [4–7]. For example, lots of studies have shown that microsatellite instability (MSI) in the genome could act as an exclusive prognostic marker in the early stages of CRC [4, 20]. Another useful tool, Septin9 hypermethylation detection in blood samples, has received researchers’ attention and was the first-approved serum test for CRC screening by FDA. But further estimation on Septin9 serum assay for CRC screening turned out that it was weakly recommended because of low sensitivity for cancer and inability to detect advanced adenomas [21]. Extensive works are still needed to provide new insights into the tumor.

CILP2 is a noncollagenous protein in human articular cartilage. In the last few years, correlations between CILP2 and plasma lipid concentration in different populations have been studied in some GWAS researches. According to Kathiresan et al. [9], in Caucasian individuals analyzed, rs16996148 variant of *CILP2* gene had a reducing role in triglyceride and LDL-C level. Rašlová et al.’s report on the association between CILP2 polymorphism and FER(HDL) supports its role in lipid metabolism [22], while in other reports, the relationship between *CILP2* polymorphism and lipid metabolism was not yet discovered [23], nor in the Japanese population [24] or Slovak Midlife women [25]. However, Luptáková et al. indicated that the minor T allele in the *CILP2* gene was associated with lower LDL-C, apoB, and atherogenic indices and higher HDL-C levels [25]. This result was by the study in the Singaporean population ranging from 40 to 80 years of age [26]. On the other hand, it has been reported that SNPs in *CILP2* gene were associated with adult height attainment [27], and CpGs in *CILP2* were statistically significantly associated with both body mass index and fat-free mass index in preschool children [28]. In summary, most of the current reports suggest the correlation between CLIP2 and lipid metabolism. However, the molecular mechanism on CLIP2 affecting lipid metabolism is still unclear. According to Zhang et al., an expression quantitative trait locus for *the CILP2* gene, rs8103992, was statistically significantly associated with adult height attainment and osteosarcoma risk after adjustment for multiple comparisons in 864 osteosarcoma cases and 1879 controls of European ancestry [16]. To our best knowledge, no more reports were describing the relationship between CILP2 and cancers.

Our work presented here has evaluated the prognostic value of CILP2 in CRC by analyzing a dataset of TCGA cohort and TMA cohort. For the first time, we found out that CILP2 was upregulated in tumor tissues compared to normal tissues. Also, we observed that CILP2 expression was statistically significantly correlated with clinicopathological parameters of CRC patients in TCGA cohort and TMA cohort. In high-stage CRC samples, CILP2 was upregulated compared to low-stage CRC samples. To evaluate the prognostic value of CILP2 on the overall survival of CRC patients in TCGA cohort, the Kaplan-Meier and Cox regression analyses were performed. We found out that higher CILP2 expression was correlated with a much poorer prognosis in the patients. These results indicated that CILP2 could act as an independent prognostic marker in CRC.

Recently, many reports have shown that obesity represents a common risk factor for several types of cancer [29, 30], especially for hormone-dependent cancers, such as breast cancer [31, 32] and advanced prostate cancer [33]. The biological association between obesity and cancer might relate to tissue lipid metabolism. It is well known that cancer cells, including CRC cells, show alterations in lipid metabolism of synthesis, desaturation, elongation, and mitochondrial oxidation of fatty acids [34–36]. A population-based study has revealed incidences of CRC to be associated with circulating levels of apolipoproteins [35]. However, there have been no reports on the specific molecular mechanism by which CLIP2 gene affects lipid metabolism, and sophisticated correlation and therapeutic use of lipid metabolism-related alternations remain further investigations.

## Conclusions

Our study has raised that CILP2 might serve as a potential prognostic marker in CRC patients. Further studies would be needed to detect CILP2 expression in serum of the patients and confirm the prognostic value and feasibility in larger and multi-center cohorts of the patients, as well as to further elucidate molecular mechanisms underlying correlations between CILP2, lipid metabolism, and CRC development.

## Data Availability

The datasets used and/or analyzed during the current study are available from the corresponding author upon reasonable request.

## Abbreviations

CILP2: Cartilage intermediate layer protein 2
TCGA: The Cancer Genome Altas
TMA: Tissue microarray
OS: Overall survival
CRC: Colorectal cancer
GWAS: Genome-wide association studies
NCAN-CILP2-PBX4: Neurocan-cartilage intermediate layer protein 2-pre-B cell leukemia homeobox 4
NAFLD: Non-alcoholic fatty liver disease
MSI: Microsatellite instability
SNPs: Single nucleotide polymorphisms
LDL-C: Low-density lipoprotein cholesterol
HDL-C: High-density lipoprotein cholesterol
